# Compound Kushen injection inhibits EMT of gastric cancer cells via the PI3K/AKT pathway

**DOI:** 10.1186/s12957-022-02609-y

**Published:** 2022-05-20

**Authors:** Luo Li, Keshan Wang, Zhenguo Liu, Yajuan Lü, Congcong Wang, Xuefei Yi, Jianping Guo

**Affiliations:** 1 Quality Management Office, Zibo Central Hospital, Zibo, 255000 Shandong China; 2 Department of Intervention, The Fourth People’s Hospital of ZiBo City, Zibo, 255000 Shandong China; 3 Department of Oncology, The People’s Hospital of Gaoqing District, Zibo, 256300 Shandong China; 4 Department of Radiotherapy, Shandong Qianfoshan Hospital, Jinan, 250014 Shandong China; 5 Department of Oncology, Maternal and Child Health Care Hospital of Zibo, Zibo, 255029 Shandong China

**Keywords:** CKI, Gastric cancer, PI3K/AKT, EMT

## Abstract

**Background:**

The effective components contained in compound Kushen injection (CKI) and the genes and signalling pathways related to gastric cancer (GC) were analyzed through the network pharmacology method of traditional Chinese medicine, and various possible mechanisms by which CKI affects the proliferation, differentiation, survival, and metastasis of GC cells were discussed. The PI3K/AKT signalling pathway is considered to be one of the most important pathways targeted by CKI in the regulation of GC cells. The implementation of related cell experiments also confirmed the information we revealed.

**Methods:**

Effective drug components of Kushen and Baituling in CKI were identified from the Traditional Chinese Medicine Systems Pharmacology Database (TCMSP). Genes related to GC were identified using the GENECARD and OMIM databases. The common target genes related to the effective components of the drug and GC were identified using the intersection method and visualized using software. A protein–protein interaction network (PPI) was established using STRING online software to confirm the key genes. Gene Ontology (GO) enrichment analysis and Kyoto Encyclopedia of Genes and Genomes (KEGG) enrichment analyses were performed to predict the key pathways of CKI in GC treatment. BGC-803 and MKN-28 GC cells were used to verify the signalling pathway. Cell proliferation, apoptosis, migration ability, and invasion ability were assessed using CCK8, flow cytometry, scratch, and transwell assays. Immunofluorescence assays and western blotting were used to detect the expression of related proteins.

**Results:**

CKI regulated GC cells through 35 effective drug components of GC-related target genes. In total, 194 genes were common targets of CKI and GC. The most significant function of the enriched genes was DNA-binding transcription activator activity as demonstrated by GO enrichment analysis. The metabolic pathway with the highest enrichment was the PI3K/AKT signalling pathway as demonstrated by KEGG enrichment analysis. Our cell experimental evidence also shows that CKI inhibits GC cell growth and migration and induce GC cell apoptosis. In addition, CKI inhibits the EMT process in GC cells through the PI3K/AKT signalling pathway.

**Conclusion:**

AKT1 is a key gene for CKI treatment of GC. CKI inhibited GC cell growth and migration and induced GC cell apoptosis. In addition, CKI regulated the EMT process in GC cells through the PI3K/AKT signalling pathway.

## Introduction

Gastric cancer (GC) is one of the most common malignancies worldwide. Statistics from relevant cancer research institutions show that there are approximately 1 million new cases of GC worldwide. GC has the second highest incidence and mortality rate among all cancers [1, 2]. The World Health Organization declared GC a public health concern. GC is characterized by a high incidence, metastasis, mortality, low early diagnosis rate, radical resection rate, and 5-year survival rate [3]. East Asian countries have the highest incidence of GC. The annual incidence rate per 100,000 inhabitants is between 40 and 60 years [4]. The detection rate of early GC is low given the lack of obvious symptoms and signs. Most patients (> 70%) develop advanced-stage GC. Some patients are not eligible for surgical resection. Latent distant metastases may be present in advanced-stage GC; therefore, the overall prognosis is poor. In recent years, many studies have been conducted to improve the prognosis of patients with GC. Therapies, such as neoadjuvant chemotherapy, radiotherapy, and molecular targeted therapy, have become effective methods to improve prognosis [5].

Traditional Chinese medicine (TCM) alleviates uncomfortable symptoms, reduces the toxicity and side effects of chemoradiotherapy, and improves the quality of life, and a number of studies have also demonstrated that TCM can improve the long-term efficacy of a variety of cancers [6–9]. There are many components of TCM, and different components have different pharmacological effects. The mechanism of action is very complex, and the modulation of various regulatory signal targets is considered to be the key to the anti-proliferation, anti-migration, and anti-metastasis effects of TCM [10]. The method of network pharmacological research is mainly based on the whole body and molecular level to identify the active ingredients in TCM to clarify the mechanism of action of each active ingredient and predict the target gene and pharmacological action of these components [11, 12].

Compound Kushen injection (CKI), a compound injection derived from Kushen and Baituling, has been used in anticancer therapy for more than 15 years. CKI contains a variety of pharmacologically active ingredients, such as matrine and oxymatrine, and has a variety of pharmacological activities, and CKI has demonstrated significant effects on cancer and tumors [13, 14]. Studies have confirmed the inhibitory effect of CKI on malignant tumor cells in the colon, brain, breast, and lung [10, 15]. However, studies on the effects of CKI on GC cells have rarely been reported. The purpose of this study was to explore the effects, targets, and pathways of CKI on GC cells based on network pharmacology and cell experiments.

## Materials and methods

### Part 1

#### Data collection from online platforms

The active components of Kushen and Baituling in CKI and genes related to components were collected from the Traditional Chinese Medicine Systems Pharmacology (TCMSP) database. TCMSP (https://tcmspw.com/tcmsp.php) contains 499 TCMs and the compound components of each TCM (greater than 29,000 in total). The TCMSP database also provides comprehensive evaluation data on human absorption, distribution, and metabolism properties for each compound component. The most powerful function of the TCMSP database is obtaining ideal active components and related target genes under various screening criteria. Active components of Kushen and Baituling in CKI and genes related to components were filtered out from the TCMSP database by searching the keywords “Kushen” and “Baituling.” Oral bioavailability (OB) > 30% and drug-likeness (DL) > 0.18 were used as screening criteria for components. OB represents the percentage of the oral drug dose that reaches the systemic circulation. A high OB is typically a key indicator for determining the active components of drugs. DL is a qualitative concept used in drug design to assess the “pharmacokinetic” status of prospective compounds, which helps optimize pharmacokinetics and drug properties. Genes related to GC were collected from the GeneCard and OMIM databases by searching the keyword “gastric cancer”. GeneCards (https://www.genecards.org/) is a searchable, integrative database that provides comprehensive, user-friendly information on all annotated and predicted human genes. OMIM (https://omim.org/) is a comprehensive, authoritative compendium of human genes and genetic phenotypes that are freely available and updated daily.

#### Statistics and visualization of data

The active components of Kushen and Baituling in CKI were merged into a file, and duplicates were eliminated. Genes related to active components were merged into a file, and duplicates were eliminated. Genes related to GC collected from GeneCard and OMIM were also merged into a file, and duplicates were eliminated. The common target genes related to the active components of CKI and GC were identified by the intersection method using R version 3.6.3. The R package “VennDiagram” was used in this process.

#### Construction of the drug-disease network and protein–protein interaction (PPI) network

A merged file of drugs, diseases, active components, and related genes was created using R version 3.6.3. This file and the merged file with active components of CKI and the merged file with common target genes related to active components of CKI and GC were imported into Cytoscape 3.6.1 (http://www.cytoscape.org/) software to construct a visual drug-disease network. In the network diagram, the four types of nodes are defined as drug, disease, active components, and genes. The active components of CKI and common target genes are connected by lines. The common target genes were imported into the STRING database version 11.0 (https://string-db.org/) to obtain a protein–protein interaction (PPI) network. The organism was selected as “Homo sapiens”, and the minimum required interaction score was set as “medium confidence = 0.99” during this process. The disconnected nodes were hidden in the PPI network. The STRING database searches for known protein–protein interactions and predicts protein–protein interactions. Using this database, the interaction network among proteins can be obtained, which is helpful for identifying the core regulatory genes. The top 20 proteins with the largest number of connections are presented as a histogram constructed using R version 3.6.3.

#### Gene ontology and pathway enrichment

To illustrate the role of the common target genes in gene function and signalling pathways, Gene Ontology (GO) enrichment analysis and Kyoto Encyclopedia of Genes and Genomes (KEGG) pathway enrichment analysis were performed using R version 3.6.3. Twenty GO categories with significant enrichment and 20 pathways with the most significant gene enrichment were plotted in the form of dot plots. *P* values < 0.05 and *Q* values < 0.05 were used as the screening criteria. The R packages “colorspace” and “stringi” and Bioconductor packages (http://www.bioconductor.org/), “DOSE,” “clusterProfiler,” and “pathview” were used in the process.

### Part 2

#### Cell culture and grouping

BGC-803/MKN-28 human GC cells (ScienCell Research Laboratory, San Diego, CA, USA) were purchased from Shanghai Huiying Biotechnology Co., Ltd. (Shanghai, People’s Republic of China). The human GC cell lines BGC-803/MKN-28 were cultured at 37 °C in tissue culture flasks (Corning Incorporated, Corning, NY, USA) containing Roswell Park Memorial Institute 1640 medium (Caisson Labs Incorporated; Corning, USA) supplemented with 10% fetal bovine serum (FBS; Gibco, Thermo Fisher Scientific) and 1% penicillin–streptomycin (HyClone; GE Healthcare UK Ltd., Little Chalfont, UK) in a humidified incubator containing 95% air and 5% CO_2_. The cell culture medium was replaced every day, and the specific growth status of the cells was observed every 6 h. When the cell density reached 80–90%, the cells were grouped. Each cell line was divided into three groups and cultured in 6-well or 96-well plates. The seeding density was 1 × 10^5^ cells/well in 6-well plates and 4 × 10^3^ cells/well in 96-well plates. Cells were cultured for 24 h before the addition of the drugs. The three groups included the high-concentration group (H-CKI, 100 μl CKI per ml of cell medium), low concentration group (L-CKI, 20 μl CKI and 80 μl saline per ml of cell medium), and control group (100 μl saline per ml of cell medium). The treatment time was 48 h for all assays.

#### Cell proliferation assay

Cell counting kit-8 (CCK8) assays were used to determine cell proliferation according to the manufacturer’s instructions (Beyotime; Institute of Biotechnology, Haimen, China). BGC-803/MKN-28 human GC cells were seeded into 96-well plates at a density of 4 × 10^3^ cells/well and cultured for 24 h before being divided into three groups. Each cell line was divided into the H-CKI, L-CKI, and control groups, and each group occupied five wells. The 96-well plate was placed in an incubator containing 95% air and 5% CO_2_ for 12 h, and the optical density (OD) values at 450 nm were measured using a microplate spectrophotometer (YIENDA, China). The same method was used to measure the OD values of the three cell suspension groups after incubation for 12 h, 24 h, 36 h, and 48 h. Cell growth curves were plotted using time as the horizontal axis and absorbance as the vertical axis.

#### Transwell assay

Invasion assays were performed in 24-well Transwell inserts (8 μm pore size; Chemicon®; Merck Millipore). According to the instructions, we placed the small chamber into the culture plate, added 300 μl warmed serum-free medium to the interior of the inserts, and allowed it to stand at room temperature for 1 h to rehydrate the Matrigel. We then aspirated the remaining culture medium. Three groups of BGC-803/MKN-28 human GC cells were used for the assays. Five hundred microlitres of media containing 10% FBS complete culture medium as a chemoattractant was added to the lower chamber, and 200 μl of cell suspension containing 1 × 10^5^ cells in each group was added to each upper chamber. After incubation in a humidified incubator containing 95% air and 5% CO_2_ at 37 °C for 48 h, non-invading cells and Matrigel were removed. The cells that invaded the lower filters were fixed in methanol for 30 min and stained with 0.25% crystal violet (Beyotime; Institute of Biotechnology, Haimen, China) for 20 min at room temperature. Five fields were randomly observed under a microscope (magnification, × 400), and the invaded cells were counted.

#### Western blot

The 6-well plates were kept on ice. The cells were removed from the medium and washed twice with PBS. Cell lysates (200 μl of cell lysate containing protease inhibitors (Solarbio Science & Technology Co., Beijing, China)) were added to each well. The cells were scraped with a cell scraper after 5 min, transferred to EP tubes, and placed on ice. The liquid supernatant was carefully collected after centrifugation at 12,000 r for 15 min at 4 °C. An appropriate amount of loading buffer was added according to the protein concentration as determined using a bicinchoninic acid assay kit (Solarbio Science & Technology Co, Beijing, China) and heated in boiling water for 5 min. Each protein sample was separated by 10% sodium lauryl sulfate polyacrylamide gel electrophoresis (30 μg per well) and then transferred to a polyvinylidene fluoride membrane (Solarbio Science & Technology Co, Beijing, China). The membranes were blocked with 5% non-fat milk for 1 h and primary antibodies, including E-cadherin (1:900; Abcam, Cambridge, UK), N-cadherin (1:600; Abcam, Cambridge, UK), vimentin (1:900; Abcam, Cambridge, UK), PI3K (1:1000; Abcam, Cambridge, UK), p-AKT1 (1:1000; Abcam, Cambridge, UK), and GAPDH (1:5000; Abcam, Cambridge, UK). The membranes were then incubated overnight at 4 °C with slow shaking. Diluted sheep anti-rat immunoglobulin G conjugated peroxidase was added and incubated for one hour at room temperature with slow shaking. Based on the manufacturer’s instructions, the HRP substrate luminol (Leagene Biotechnology, Beijing, China) was added to the membrane, and a FluorChem E instrument (Jinpeng Analytical Instruments Co., Ltd., Shanghai, China) was used to detect the immune signal. The greyscale values of bands were analysed using ImageJ software (version 1.80; National Institutes of Health, Bethesda, MD, USA).

#### Flow cytometry

A total of six groups of cells from the two cell lines were washed twice with cold PBS and resuspended in a 1 × 10^6^/ml cell suspension. Then, 100 μl of the cell suspension was incubated with 5 μl of FITC-Annexin V (Shanghai Yaji Biotechnology Co., Ltd., Shanghai, China) and 5 μl of propidium iodide (Shanghai Yeasen Biotechnology Co., Ltd., Shanghai, China) at room temperature in the dark for 15 min. SA-FLOUS solution was added, and the mixture was incubated at 4 °C for 20  min. Flow cytometry was used to detect the apoptosis rate. The experiment was repeated thrice, and the average value was calculated.

#### Statistical analysis

The data from the experiments were analysed using GraphPad Prism 7 software (San Diego, CA, USA). Values are expressed as the mean ± standard deviation (SD). Unpaired Student’s *t* tests were used for comparisons between the values of different groups. Statistically significant results are presented as ∗*P* < 0.05. *P* < 0.05 was considered statistically significant.

## Results

### Active components of CKI and target genes

**Fig. 1:**
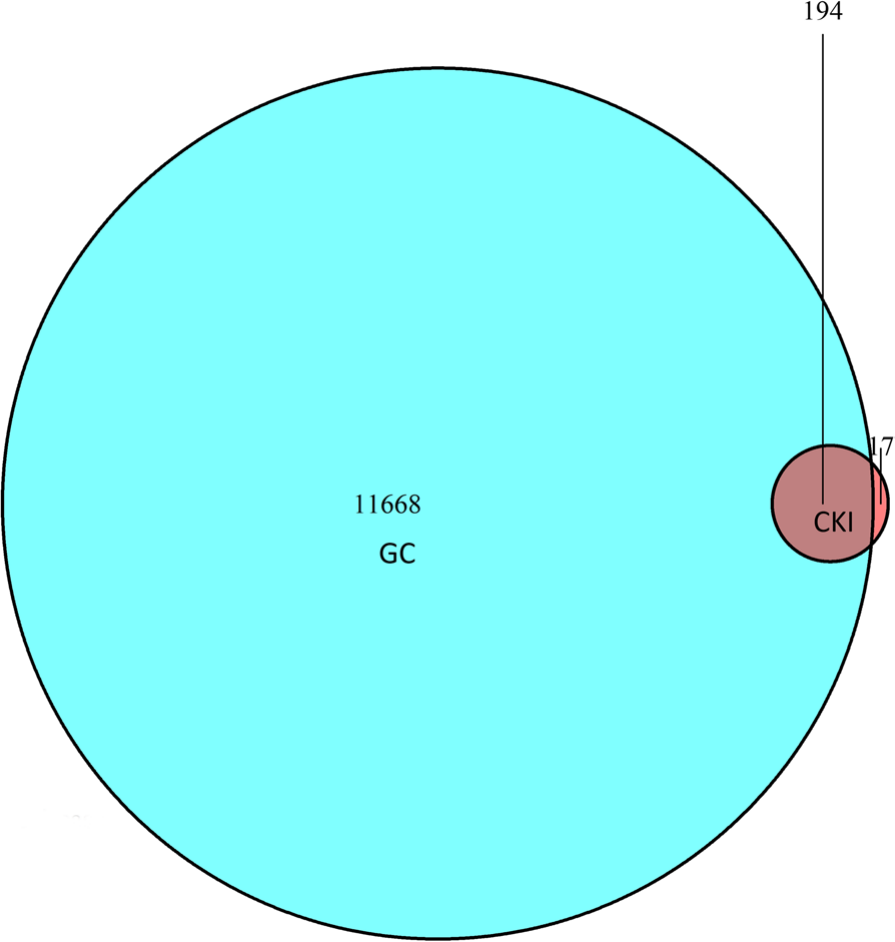
194 common target genes for GC and active components of CKI

Thirty-five active components of CKI and 211 genes were screened from the TCMSP database using OB > 30% and DL > 0.18 as screening criteria. The active components mainly include kushenin, quercetin, luteolin, stigmasterol, and formononetin. A total of 11,862 genes related to GC were collected from the GeneCard and OMIM databases. As shown in Fig. 1, 194 genes are common target genes related to GC and the active components of CKI.

### Drug-disease network and PPI network

**Fig. 2:**
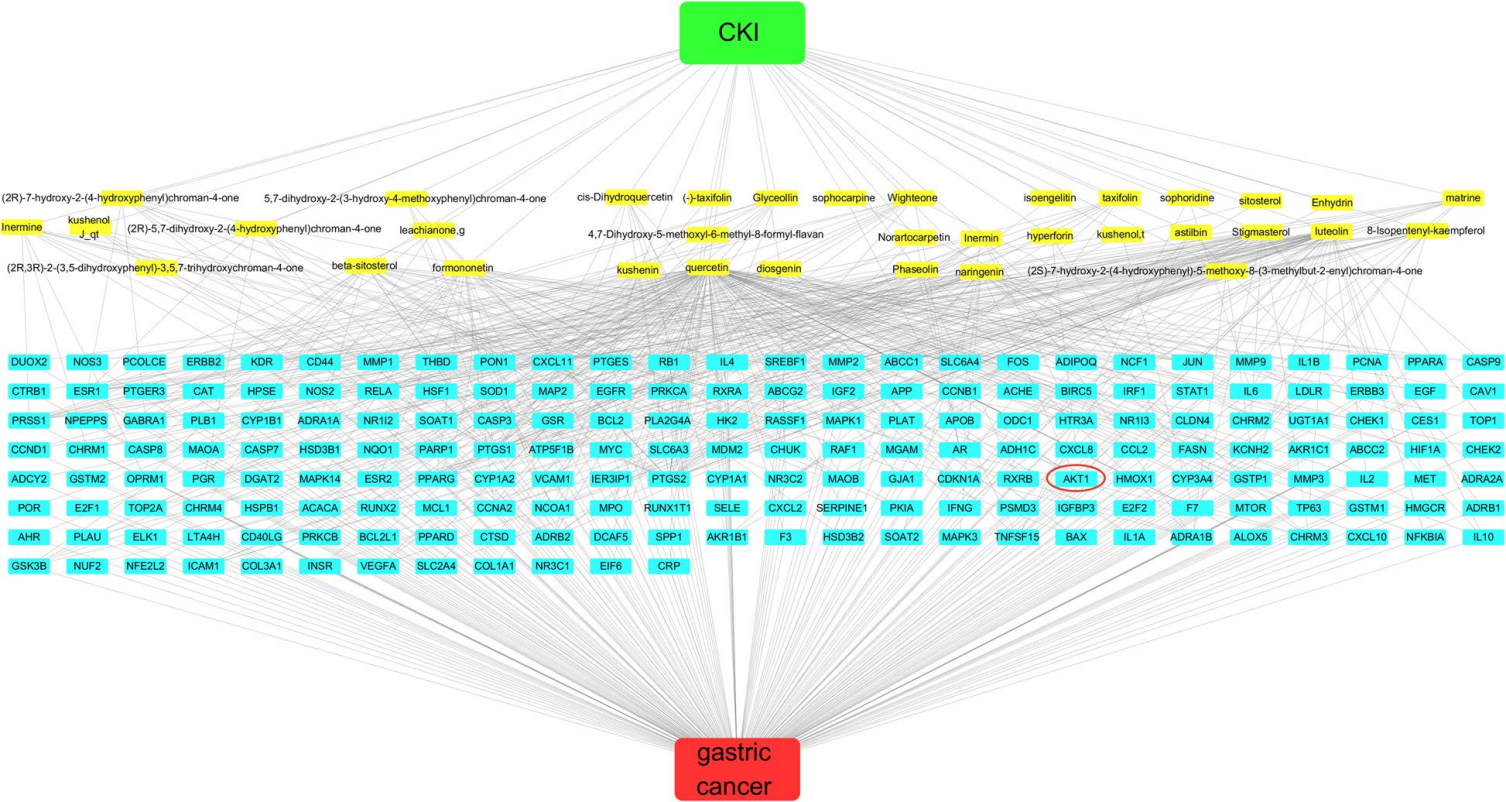
The target network diagram of GC and active ingredients of CKI. The green node represents CKI, the yellow node represents active ingredients of CKI, the blue node represents the common target gene of GC and active ingredients of CKI, and the red node represents GC. The AKT1 gene is considered the core regulatory gene and is marked with a red circle

**Fig. 3:**
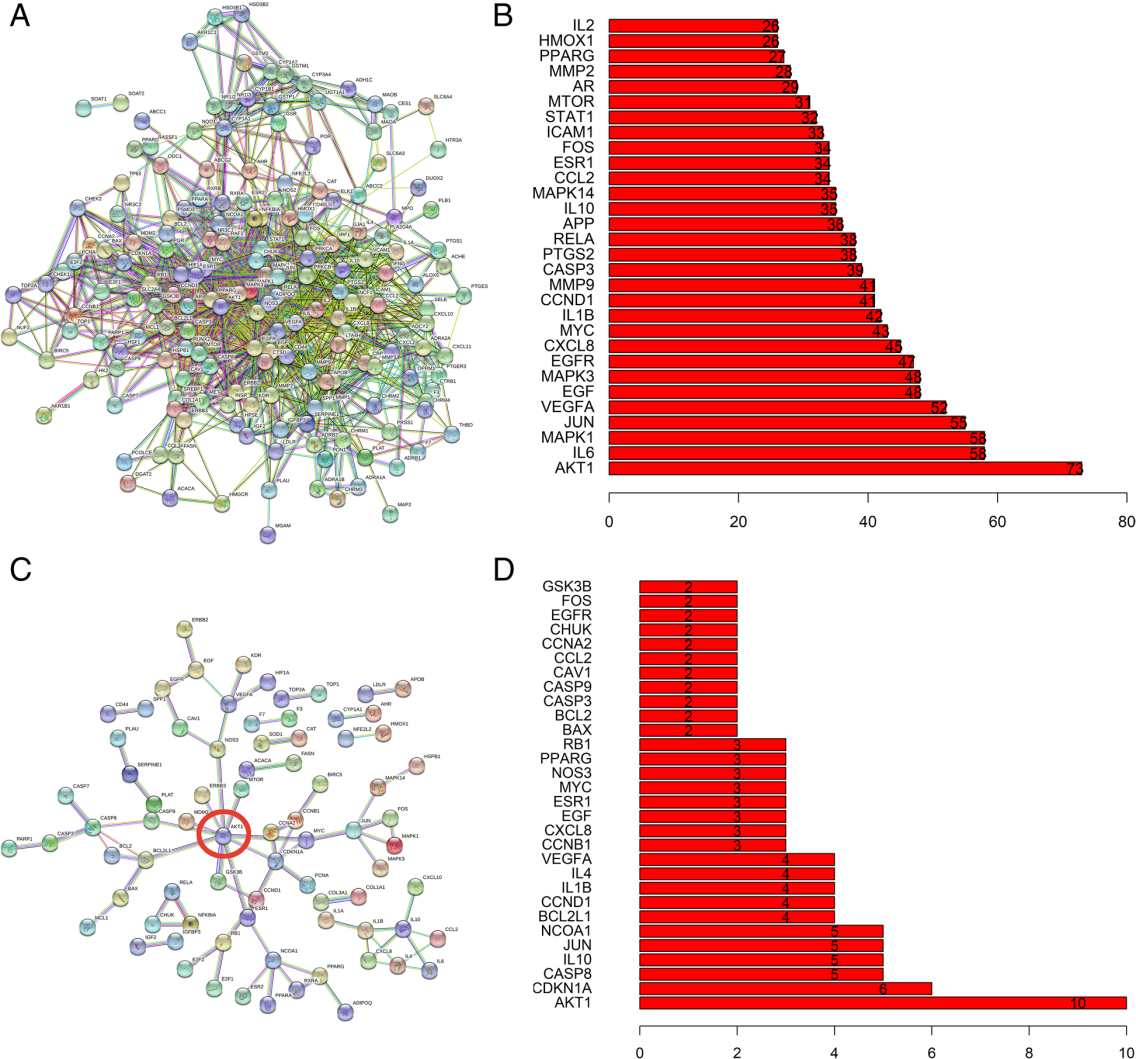
**A** PPI network diagram obtained when “median confidence = 0.40.” **B** Top 20 genes with the largest number of connections when “median confidence = 0.40.” **C** PPI network diagram obtained when “median confidence = 0.99.” The AKT1 gene has the largest number of connections and is marked with a red circle. **D** Top 20 genes with the largest number of connections when “median confidence = 0.99”

To show the connection between CKI and GC, a visual drug-disease network (Fig. 2) was constructed using Cytoscape 3.6.1 (http://www.cytoscape.org/) software. There were 231 nodes with four attributes in the diagram. These four attributes include disease, drug, active components, and target genes. The related nodes are connected by lines. The number of lines for each node often represents the importance of nodes. The top ten active components of CKI with the most associated target genes were kushenin, quercetin, luteolin, stigmasterol, formononetin, diosgenin, 8-isopentenyl-kaempferol, inermine, matrine, and phaseolin. The STRING database (version 11.0) was used to build a network of target proteins. The common target genes were imported into the “Protein name” box, and the organism was selected as “*Homo sapiens*”. The minimum required interaction score was set as “medium confidence = 0.40” during this process. In the PPI network, each node represents a protein, and the connection between nodes represents the interaction between proteins. The PPI network is shown in Fig. 3. The top 20 genes with the largest number of connections are presented in the form of a histogram in Fig. 3. A simplified PPI network is also shown in Fig. 3. When the minimum required interaction score was set as “medium confidence = 0.99,” the purpose of the simplified PPI network was to clearly show the key proteins identified. AKT1 is a key protein in the network. Other key proteins include IL6, MARK1, JUN, and VEGFA.

### Gene ontology and pathway enrichment

**Fig. 4:**
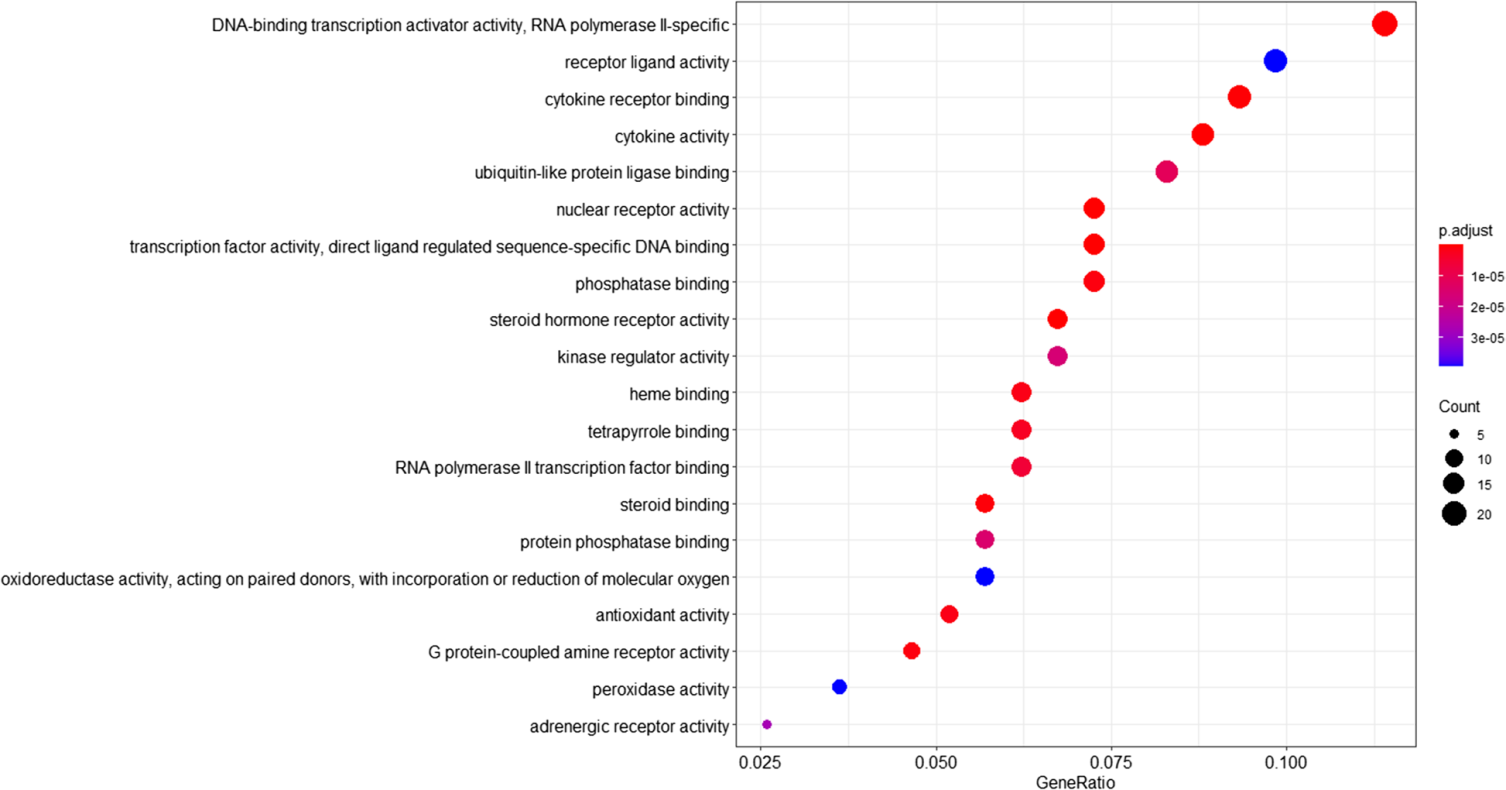
Result of GO enrichment analysis, the size of the dots represents the number of genes under different GO categories, and the different colours of the dots represent the change in the adjusted *P* value

**Fig. 5:**
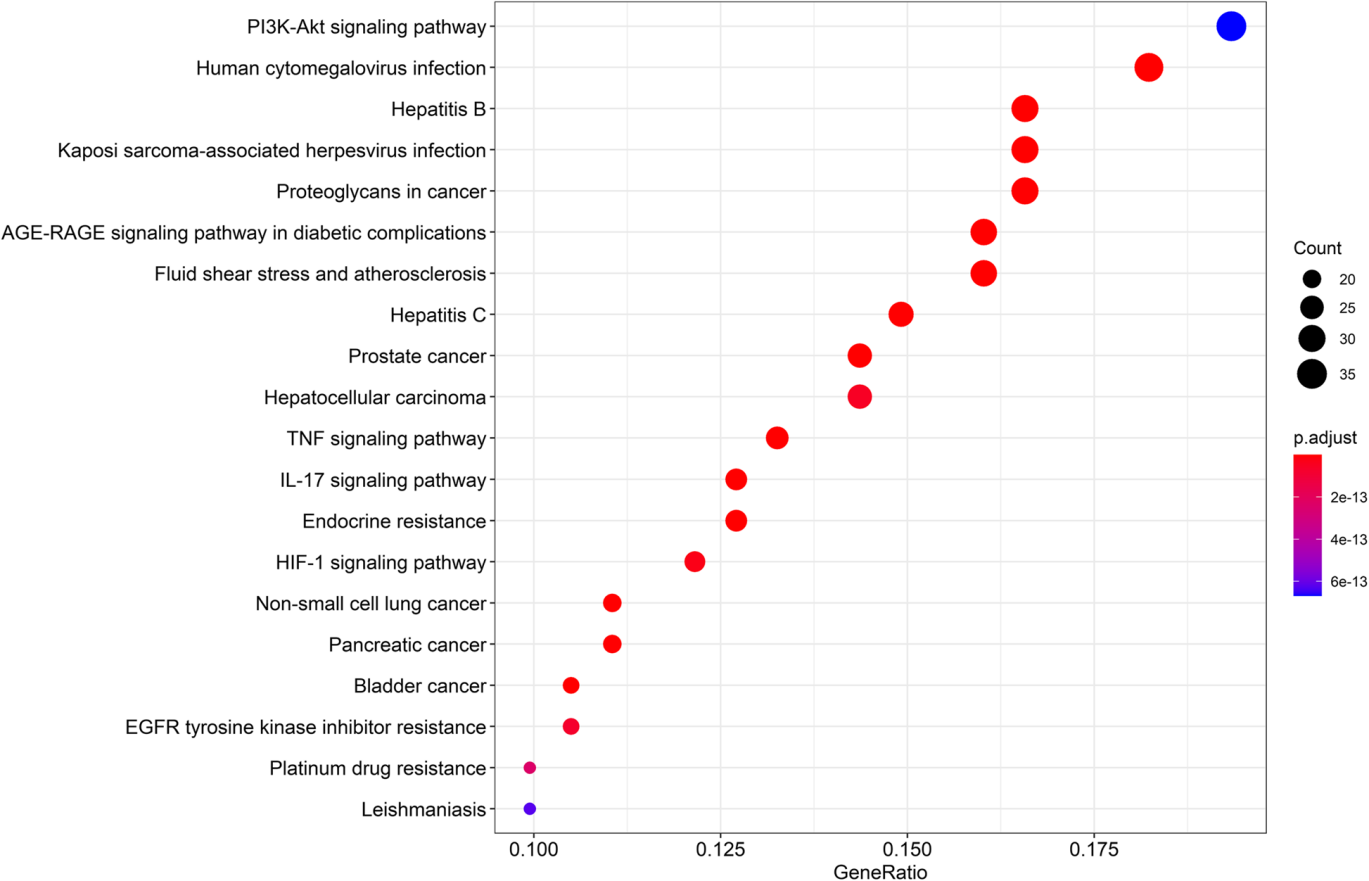
Result of KEGG signalling pathway enrichment analysis. The size of the dots represents the number of genes under different KEGG pathways, and the different colours of the dots represent the change in the adjusted *P* value

**Fig. 6:**
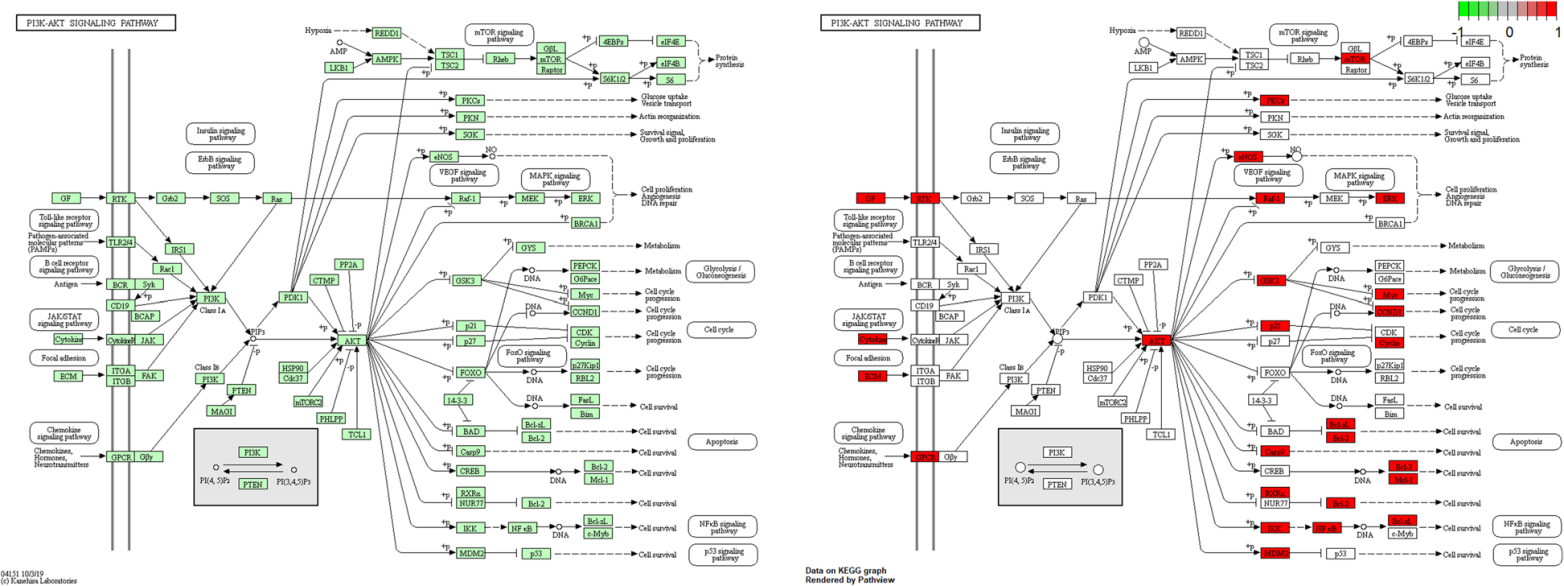
Diagram of the PI3K/AKT signalling pathway. Blue nodes indicate all nodes in the PI3K/AKT signalling pathway, and red nodes are the sites where CKI active ingredients act on the PI3K/AKT signalling pathway

A total of 129 GO categories were obtained by GO enrichment analysis using R version 3.6.3. The top 20 GO categories with significant enrichment are plotted in the form of dot plots (Fig. 4). In the dot plot, the size of the dots represents the number of genes under different GO categories, and the different colors of the dots represent the change in the adjusted *P* value. A total of 172 KEGG pathways were identified through KEGG pathway enrichment analysis using R version 3.6.3. The top 20 KEGG pathways with significant enrichment are plotted in the form of dot plots (Fig. 5). In the dot plot, the size of the dots represents the number of genes under different KEGG pathways, and the different colors of the dots represent the change in the adjusted *P* value. KEGG pathway enrichment analysis results reveal that the PI3K/AKT signalling pathway is the pathway with the most enriched genes. The relevant genes of the active ingredients of CKI in the PI3K/AKT signalling pathway are shown in the form of a pathway diagram (Fig. 6). We hypothesized that the PI3K/AKT signalling pathway is the most important pathway by which CKI acts on GC. Through this pathway, CKI regulates the EMT phenomenon in GC cells.

### CKI inhibits GC cell proliferation

**Fig. 7:**
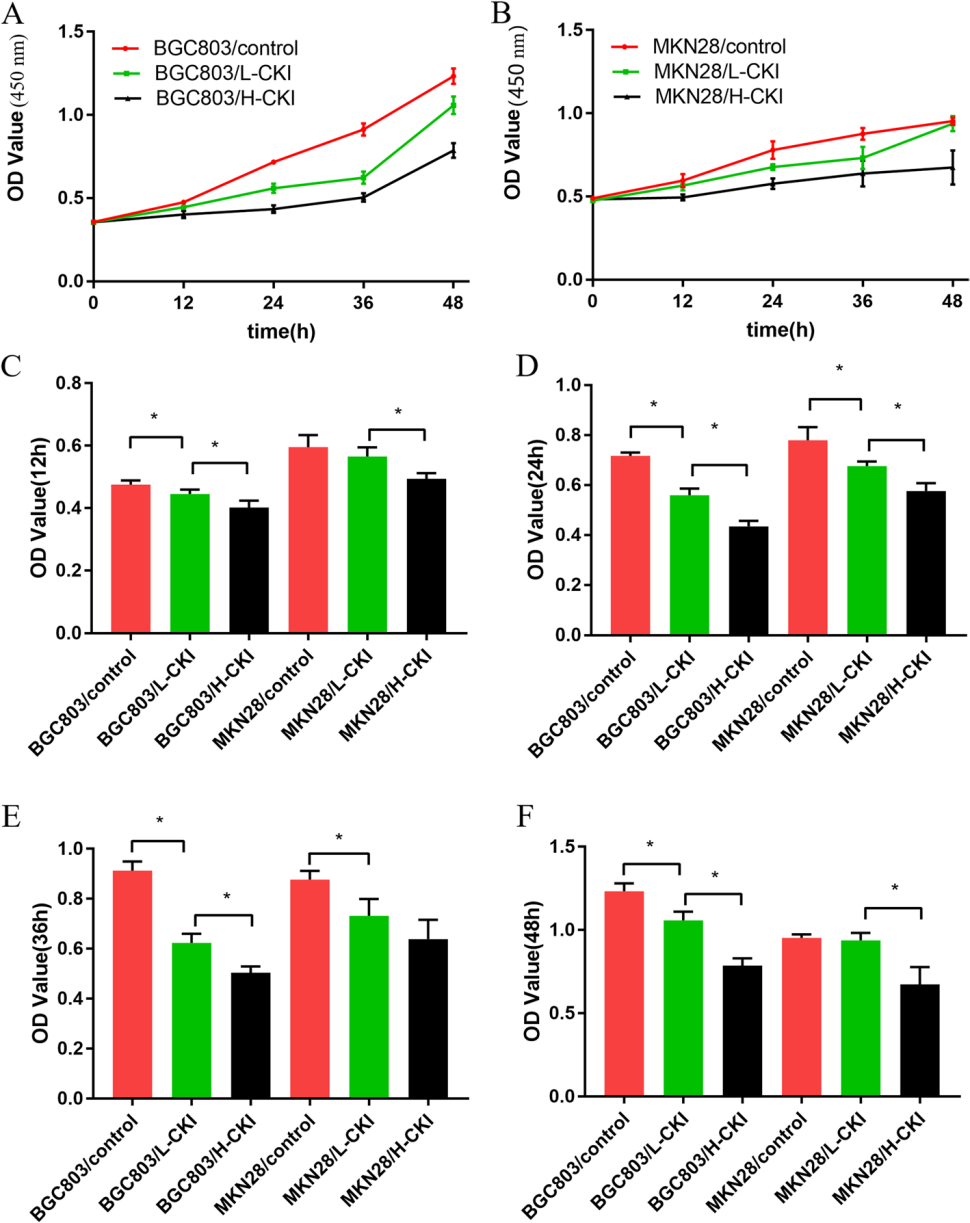
Comparison of GC cell proliferation results in each group of GC cells revealed the following trend: Control>L-CKI>H-CKI. **A** Graph of the proliferation of BGC803 cells treated with different CKI concentrations. **B** Graph of MKN28 cell proliferation under the action of different CKI concentrations. **C** Comparison of the absorbance of two types of cells under the action of different CKI concentrations at 12 h. **D** Comparison of the absorbance of two types of cells under the action of different CKI concentrations at 24 h. **E** Comparison of the absorbance of two types of cells under the action of different CKI concentrations at 36 h. **F** Comparison of the absorbance of two types of cells under the action of different CKI concentrations at 48 h. Statistically significant differences are shown as *P < 0.05

CCK-8 analysis was used to evaluate the effect of CKI on BGC-803 and MKN-28 cell proliferation. Under CKI stimulation, the OD values at 450 nm of the two types of GC cells decreased significantly. With an increasing CKI concentration, the OD values at 450 nm of the two types of GC cells decreased more significantly, indicating that CKI affected the proliferation rate of the two types of GC cells, and this effect increased with increasing CKI concentration. The CCK8 experimental results of the six groups of BGC-803 and MKN-28 cells are shown in Fig. 7.

### CKI inhibits gastric cancer cell invasion

**Fig. 8:**
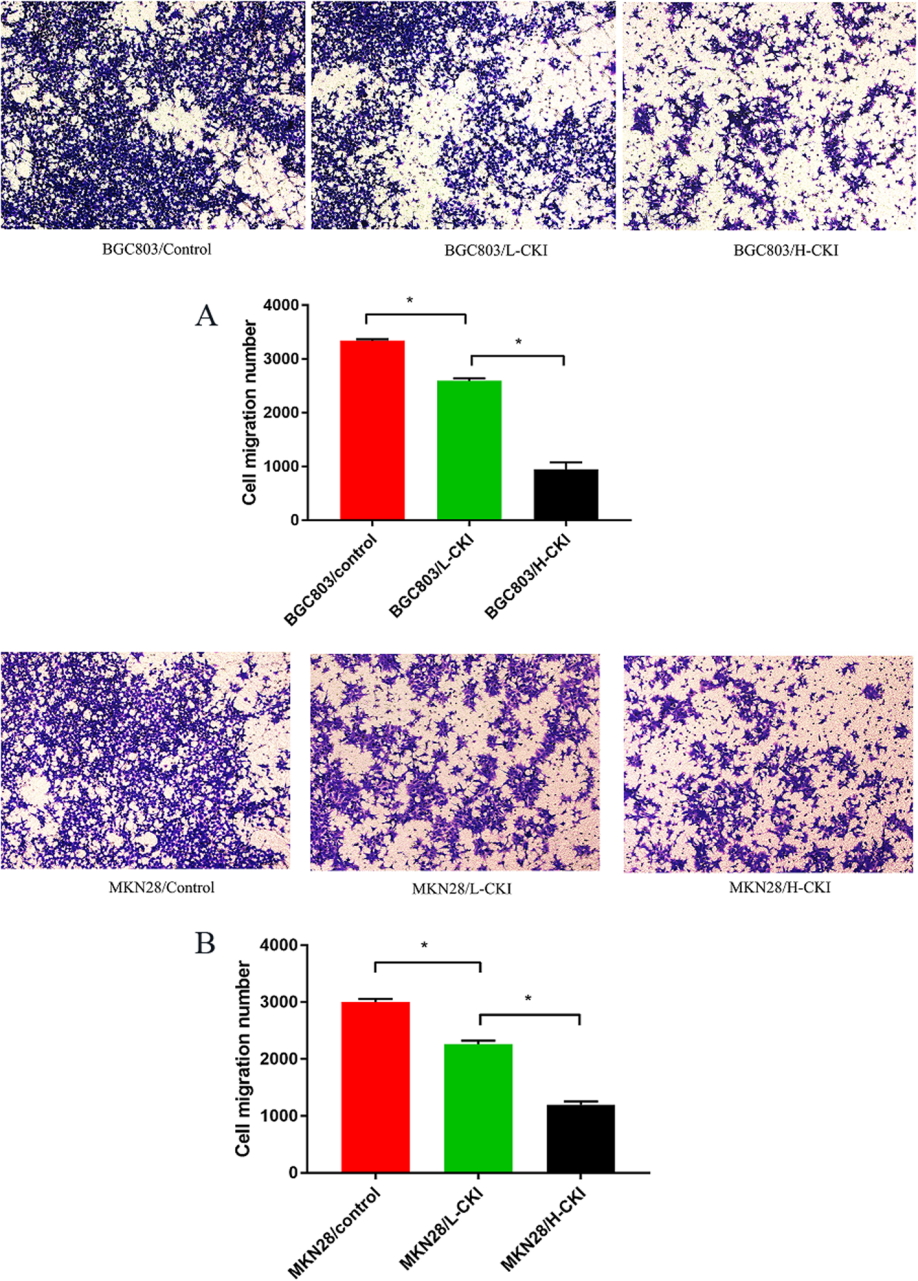
The comparison of GC cell invasion rate in each group revealed the following trend: Control>L-CKI>H-CKI. **A** Comparison of the number of invasive BGC803 cells in each group. **B** Comparison of the number of invasive MKN28 cells in each group. Statistically significant differences are shown as *P < 0.05

Transwell migration analysis was used to evaluate the inhibitory effect of CKI on GC cell invasion. A comparison of the number of invasive cells in each group of GC cells penetrating the matrix membrane is shown in Fig. 8. Comparison of the number of invasive BGC803 and MKN28 cells in the three groups revealed the following trend: Control>L-CKI>H-CKI. CKI significantly inhibited GC cell invasion, and this effect increased with an increase in CKI concentration.

### CKI inhibits EMT in GC cells

**Fig. 9:**
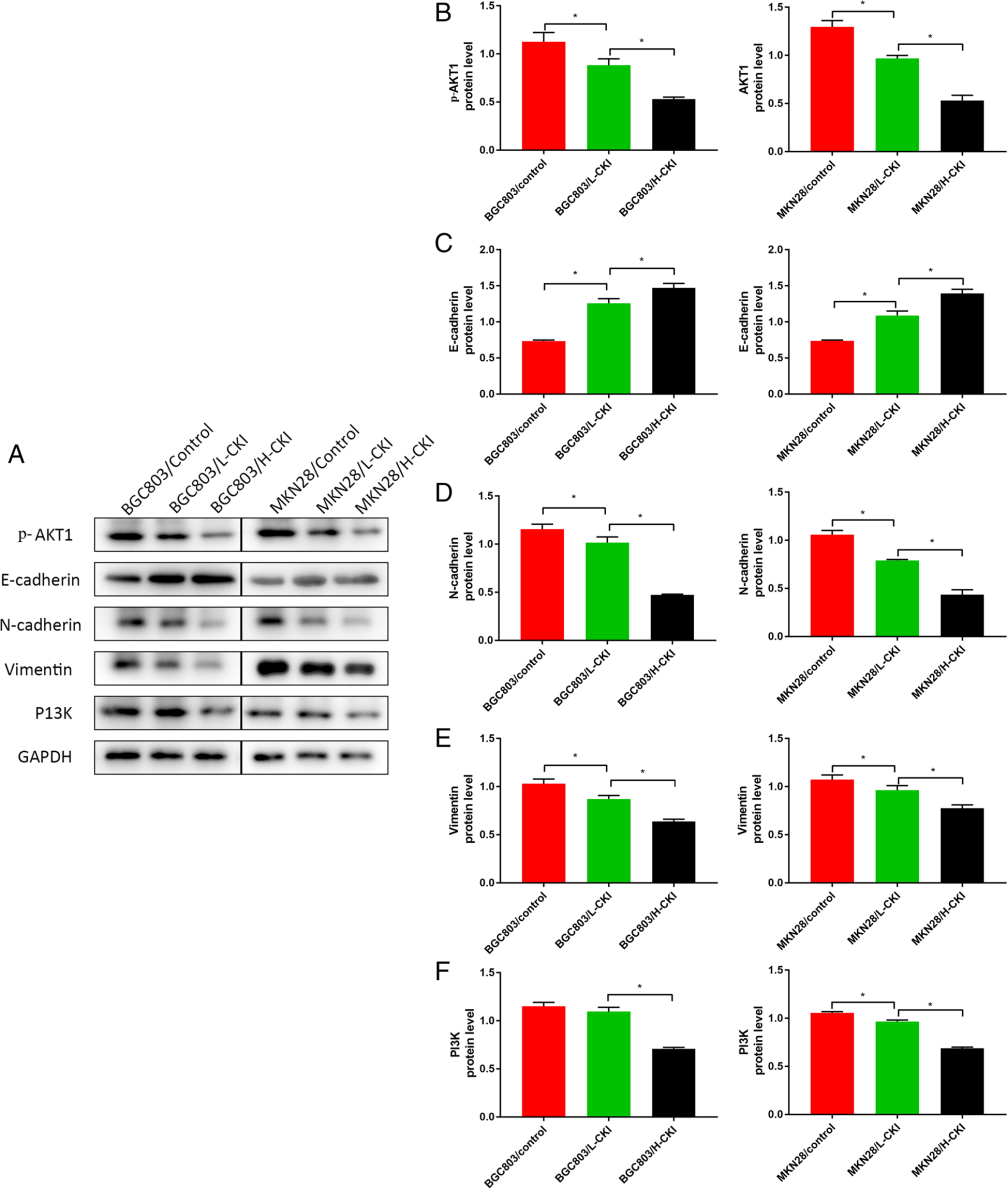
**A** Protein expression levels of p-AKT1, E-cadherin, N-cadherin, vimentin, and PI3K in BGC803 cells and MKN28 cells. **B** Comparison of p-AKT1 expression in each group revealed the following trend: Control>L-CKI>H-CKI. **C** Comparison of E-cadherin expression in each group revealed the following trend: Control<L-CKI<H-CKI. **D** Comparison of N-cadherin expression in each group revealed the following trend: Control>L-CKI>H-CKI. **E** Comparison of Vimentin expression in each group revealed the following trend: Control>L-CKI>H-CKI. **F** Comparison of PI3K expression in each group revealed the following trend: Control>L-CKI>H-CKI. Statistically significant differences are shown as *P < 0.05

Western blot results from a total of six groups of two GC cell lines showed that the expression of E-cadherin increased significantly, and the expression of vimentin and N-cadherin decreased significantly under the action of CKI. E-cadherin, N-cadherin, and vimentin are typical hallmarks of epithelial and mesenchymal phenotypes. Therefore, significant inhibition of EMT in GC cells occurred under the action of CKI, and this effect increased with increasing CKI concentration. Western blot results also showed that p-AKT1 and PI3K expression decreased significantly under the action of CKI, and this effect also increased as the CKI concentration increased. This finding demonstrates that CKI is likely to inhibit the EMT of gastric cancer cells through the PI3K/AKT pathway. Western blot results are shown in Fig. 9.

### CKI increases the GC cell apoptosis rate

**Fig. 10:**
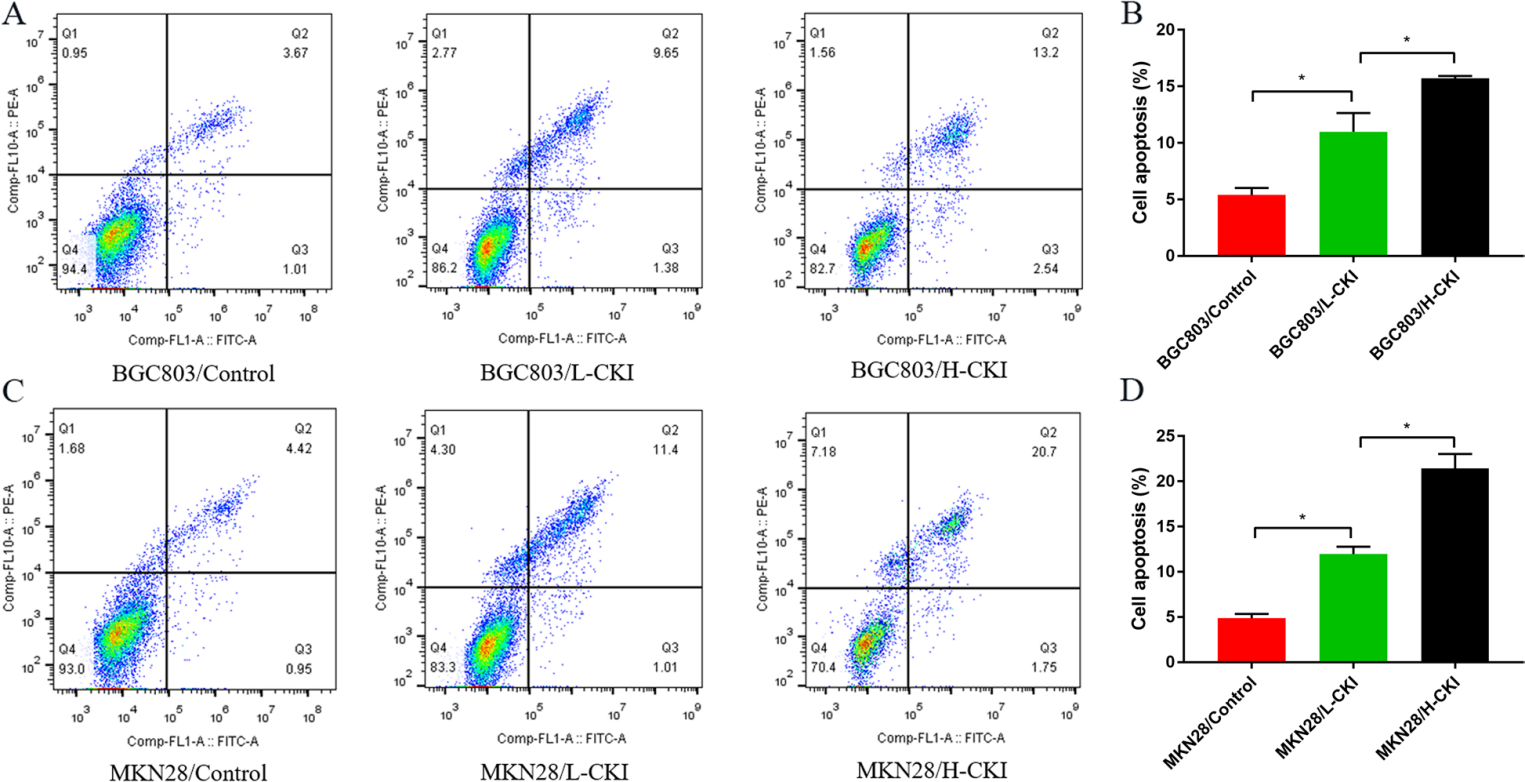
**A** Apoptosis results of BGC803 cells in each group. **B** Comparison of the apoptosis rate of BGC803 cells in each group revealed the following trend: Control<L-CKI<H-CKI. **C** Apoptosis results of MKN28 cells in each group. **D** Comparison of the apoptosis rate of MKN28 cells in each group revealed the following trend: Control<L-CKI<H-CKI. Statistically significant differences are shown as *P < 0.05

The results of flow cytometry showed that the apoptosis rates of the BGC803/Control, BGC803/L-CKI, and BGC803/H-CKI groups were 5.40 ± 0.63%, 10.99 ± 1.65%, and 15.70 ± 0.22%, respectively. The apoptosis rates of the MKN28/control, MKN28/L-CKI, and MKN28/H-CKI groups were 4.88 ± 0.46%, 11.98 ± 0.79%, and 21.42 ± 1.61%, respectively. Comparison of apoptosis rates of BGC803 and MKN28 cells in the three groups revealed the following trend: Control<L-CKI<H-CKI. Statistical analysis is shown in Fig. 10.

## Discussion

GC is a disease caused by many factors, such as the environment, genes, and infection. Given that early GC is difficult to detect, its prognosis is usually poor, and the 5-year survival rate is less than 20% [16]. A variety of treatment methods have been applied to different stages of gastric cancer, such as endoscopic submucosal dissection, surgery, chemotherapy, and radiotherapy [17–20]. However, treatment measures, such as surgery, radiotherapy, and chemotherapy, often have adverse effects on the human body and even endanger the lives of patients [21, 22]. Therefore, it is necessary to identify treatment measures with fewer adverse effects based on traditional treatments.

Traditional Chinese medicine offers great advantages in treating cancer and improving the quality of life of patients [23]. As an important treatment method in traditional Chinese medicine, Chinese medicine injection has been widely used in cancer treatment, and its efficacy has been demonstrated. CKI is a Chinese medicine injection extract from Kushen and Baituling. The main components of Kushen and Baituling have a variety of pharmacological effects, such as anti-inflammatory, antiviral, immunomodulatory, and anticancer effects [24–28]. In terms of anticancer effects, CKI inhibits the invasion and metastasis of various cells, including colon, brain, and breast cancer cell lines, in vitro [10]. In vivo analyses showed that CKI inhibits the growth of MCF-7 stem-like SP cells and rat gastric carcinogenesis [29, 30]. CKI has also been widely used in clinical practice for many years, and its efficiency has been demonstrated [31–33].

In this study, we constructed a drug-active component-target gene-GC network and a PPI network of GC to systematically analyse the mechanism of CKI in the treatment of GC. Thirty-five active components of CKI, including quercetin, matrine, naringenin, and luteolin, are regarded as the main components in the treatment of GC. Quercetin has a variety of biological effects, including inhibition of the proliferation of various cancer cells and participation in anticancer signal transduction pathways [34]. Matrine has been shown to exhibit antitumor effects in different cancer cells in vitro [35]. A study showed that the expression of inhibitor of κB kinase β (IKKβ) was downregulated by matrine via the NF-κB signalling pathway in breast cancer cells [36]. Another study indicated that matrine may induce breast cancer cell (MCF-7) apoptosis and cause cell cycle arrest in the G1/S phase through the miR-21/PTEN/AKT pathway [37]. Naringenin promotes apoptosis signal-regulation kinase 1 (ASK1)-induced apoptosis via reactive oxygen species (ROS) in human pancreatic cancer cells [38]. Luteolin inhibits colorectal cancer cell metastasis by regulating the miR384/pleiotrophin axis [39]. We believe that multiple active components can regulate the proliferation, differentiation, and metastasis of GC cells through multiple pathways. PPI network analysis showed that the main proteins through which CKI acts on gastric cancer cells include AKT1, IL6, MAPK1, and JUN. AKT1 is an isoform of AKT [40], and amplification of Akt1 occurs at a higher rate in various cancers, such as gastric, breast, colon, esophageal, ovarian, pancreatic, and thyroid cancers and glioblastoma [41–44]. AKT is a serine/threonine kinase that plays a key role in many signalling pathways, including the PI3K/AKT signalling pathway. AKT is activated by a wide range of growth signals and modulates the functions of many downstream proteins involved in cellular survival, proliferation, migration, metabolism, and angiogenesis. AKT is frequently deregulated in many types of human cancers [45]. AKT is often overexpressed in human cancers. Studies have confirmed that aberrations of AKT trigger the occurrence and development of tumours and cause many types of cancer to develop resistance to conventional chemotherapy [46]. IL6 is a pleiotropic cytokine that can induce the epithelial-mesenchymal transition (EMT) of cancer cells, thereby enhancing the invasiveness and distant metastasis ability of cancer cells. The expression of IL6 in the blood and tissues of patients with cancer is significantly increased [47]. MAPK1 and JUN are important nodes in the MAPK1 pathway. By upregulating the activity of TP53 and TNFSF10 and downregulating the JUN gene, MAPK1 can be inhibited and induce apoptosis in leukaemia cells [48]. The central role of AKT in a wide range of tumours makes it an excellent therapeutic target for the treatment of different tumours [40]. We believe that AKT is the most important target of CKI for the treatment of GC.

We performed GO analysis to clarify the biological and molecular functions of these genes involved in CKI acting on GC and found that these genes are highly related to DNA-binding transcriptional activator activity, RNA polymerase II specific, receptor ligand activity, cytokine receptor binding, and cytokine activity. Through KEGG analysis, we believe that these genes mainly affect GC cells through multiple signalling pathways, and the PI3K/AKT signalling pathway may be one of the most important pathways. On the one hand, KEGG analysis showed that these genes involved in CKI acting on GC have the highest degree of enrichment in the PI3K/AKT signalling pathway. On the other hand, AKT1 is the dominant gene in the PPI network, and the conventional pathway in which AKT plays a major oncogenic role is the PI3K/AKT signalling pathway. The PI3K/AKT signalling pathway plays an important role in cancer proliferation, invasion, and metastasis and is one of the most important signalling pathways in cancer [49–52]. PI3K initiates this signalling pathway and activates AKT, leading to cell resistance to apoptosis and promotion of cell proliferation and survival [53]. The PI3K/AKT signalling pathway is also involved in the resistance of cancer cells to radiotherapy and chemotherapy [54, 55]. Some studies have confirmed that matrine, as one of the main components of CKI, inhibits the proliferation and metastasis of GC cells through the PI3K/AKT signalling pathway [56], and our experiments further confirmed this result to some extent.

EMT refers to the process by which epithelial cells lose polarity and transform into mesenchymal cells under certain conditions. Its biological characteristics include the reduced or complete disappearance of intercellular adhesion, loss of polarity, and enhanced cell motility, invasion, and metastasis. Through EMT, cells reduce or lose epithelial cell markers, such as E-cadherin, cytokeratin, and occluding, and increase or acquire mesenchymal cell markers, such as N-cadherin, vimentin, and fibronectin. The reduction or loss of E-cadherin encoded by the CDH1 gene is the most important landmark change in EMT. Studies have found that EMT is closely associated with tumour chemotherapy resistance [57]. Western blot results showed that CKI significantly increased the expression of E-cadherin and decreased the expression of N-cadherin and vimentin in GC cells, and this phenomenon became more obvious as the concentration of CKI increased. The transwell assay further confirmed that CKI could decrease the invasive ability of GC cells, and the effect of CKI was related to the CKI dose. Evidence shows that CKI can significantly inhibit the EMT of GC cells, and this effect becomes more obvious as the dose of CKI increases. In addition, we observed that P13K and p-AKT1 expression also decreased significantly and became more significant with an increase in CKI concentration, indicating that CKI has a significant inhibitory effect on the P13K/AKT pathway. The P13K/AKT pathway is also highly correlated with EMT in many tumors [58, 59]. Therefore, we hypothesized that the PI3K/AKT signalling pathway is the most important pathway for CKI to act on GC. Through this pathway, CKI regulates the EMT phenomenon in GC cells.

In our study, the cell proliferation assay and flow cytometry results showed that CKI inhibits GC cell proliferation and promotes apoptosis. These functions are related to the dosage of CKI, and the effect becomes more obvious as the dosage of CKI increases. We hypothesized that these experimental results are related to the regulation of the P13K/AKT signalling pathway by CKI. Of course, we also acknowledge the possibility that CKI regulated other signalling pathways in the context of GC. Both our data analysis results and other studies have shown that CKI has multiple pathways that regulate GC cells, including the MAPK signalling pathway, TNF signalling pathway, IL-17 signalling pathway, and P53 signalling pathway [60]. These signalling pathways influence each other to form a complex network of effects. The impact of CKI on GC that occurs through other pathways requires further study.

## Conclusion

In conclusion, GC cell proliferation and invasion was significantly reduced, and the EMT of GC cells was inhibited under the action of CKI. Furthermore, these effects are related to the concentration of CKI. These effects of CKI on GC cells may be mediated through the PI3K/AKT pathway. Our data may further demonstrate the potential value of CKI, promote the application of the drug, and support the use of CKI as a promising adjuvant therapy for antitumor therapy.

## Data Availability

The datasets used and/or analyzed during the current study are available from the corresponding author on reasonable request.
